# Prenylcysteine Oxidase 1 Deficiency Protects the Cardiac Muscle Cell Line HL‐1 Against Ischaemic/Hypoxic Stress

**DOI:** 10.1096/fj.202502993R

**Published:** 2026-04-20

**Authors:** Cristina Banfi, Lisa Brocca, Alice Bascucci, Giulia Giusy Papaianni, Alice Mallia, Sonia Eligini

**Affiliations:** ^1^ Unit of Functional Proteomics, Metabolomics, and Network Analysis Centro Cardiologico Monzino IRCCS Milan Italy

**Keywords:** cardiomyocytes, cell death, HL‐1, ischaemic/hypoxic stress, prenylcysteine oxidase 1

## Abstract

Loss of cardiomyocytes during hypoxia–reoxygenation injury contributes to adverse myocardial remodeling, resulting in hypertrophy of surviving cardiomyocytes, interstitial fibrosis, and ultimately, heart dysfunction. Despite extensive research in the field, there is currently no specific treatment available for myocardial hypoxia–reoxygenation injury to prevent cardiomyocyte death. Prenylcysteine oxidase 1 (PCYOX1) is a pro‐oxidant, FAD‐dependent thioether oxidase that generates hydrogen peroxide during prenylcysteine metabolism, but its role in cardiomyocytes is poorly defined. Here, using HL‐1 cardiomyocytes stably silenced for *Pcyox1*, we show that PCYOX1 contributes to both basal and stress‐induced oxidative burden and cell death. *Pcyox1* silencing reduced reactive oxygen species (ROS) levels at baseline and blunted the ROS increase during ischaemic/hypoxic stress. Consistently, *Pcyox1* silencing decreased apoptosis after prolonged ischaemic/hypoxic exposure. Quantitative proteomics of whole‐cell lysates and isolated mitochondria revealed coordinated remodeling of pathways involved in energy buffering and contractile machinery, including increased abundance of mitochondrial creatine kinases (CKMT1/CKMT2), acetyl‐CoA synthetase 2‐like (ACSS1), and multiple myosin components, changes that persisted under ischaemic/hypoxic stress and after reoxygenation. Overall, these data identify PCYOX1 as a modulator of redox homeostasis and proteomic adaptation in cardiomyocytes and support PCYOX1 inhibition as a potential strategy to limit hypoxia–reoxygenation–associated injury.

## Graphical abstract

Ischaemic/hypoxic stress leads to heart remodeling and dysfunction. PCYOX1 promotes cardiomyocyte survival during injury, reducing the generation of reactive oxygen species. PCYOX1 deficiency regulates key proteins to preserve cardiomyocyte structure and function such as creatine kinase.

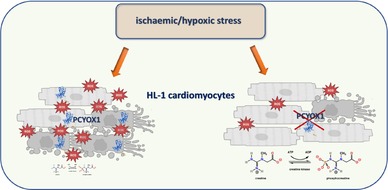

## 1 Introduction

Acute myocardial infarction (MI) remains a leading cause of death and long‐term disability worldwide [1]. Early reperfusion, achieved by primary percutaneous coronary intervention or thrombolysis, is the cornerstone of therapy to limit acute ischaemic injury and reduce infarct size. However, reperfusion itself can exacerbate cardiomyocyte loss through the multifactorial process of ischaemia–reperfusion injury, thereby contributing to adverse remodeling and increasing the risk of post‐MI heart failure [2, 3]. Among the mechanisms implicated, oxidative stress is regarded as a central determinant of cardiomyocyte death during ischaemia and after reperfusion; when excessive or persistent, reactive oxygen species (ROS) also promote maladaptive remodeling, heart failure, and aging‐related cardiomyopathy [4, 5]. Because ROS act as both signaling mediators and effectors of damage, cardiomyocyte redox homeostasis must be tightly controlled; identifying factors that shift this balance is therefore a necessary step toward reducing injury and preserving function [6]. The myocardium harbors multiple ROS‐generating systems. Mitochondria constitute a major source of ROS, and additional enzymatic contributors include xanthine oxidoreductase and nicotinamide adenine dinucleotide phosphate (NADPH) oxidases (reviewed in Ref. [7]). Despite this, the repertoire of pro‐oxidant enzymes that can amplify ROS burden in cardiomyocytes is likely incomplete. We recently identified prenylcysteine oxidase 1 (PCYOX1) as a pro‐oxidant enzyme involved in atherosclerosis development [8]. PCYOX1 is a flavin adenine dinucleotide (FAD)–dependent thioether oxidase implicated in the catabolism of prenylcysteines generated during turnover of prenylated proteins, and its catalytic activity produces a stoichiometric amount of hydrogen peroxide (H_2_O_2_) [9, 10, 11]. Consistent with this biochemical property, we previously showed that PCYOX1 contributes to cellular H_2_O_2_ production [8], and promotes oxidative modifications when associated with lipoproteins [12]; moreover, *Pcyox1* deficiency reduced lipid peroxidation in vivo [8]. However, whether PCYOX1 contributes to cardiomyocyte redox imbalance and survival during ischaemic‐like stress remains undefined. In the present study, we addressed this gap using HL‐1 cardiomyocytes, an established in vitro model for cardiac stress responses. By generating HL‐1 cells with stable *Pcyox1* silencing, we tested the hypothesis that PCYOX1 contributes to basal and stress‐induced oxidative burden and thereby promotes cardiomyocyte death during hypoxia–reoxygenation. We show that loss of PCYOX1 enhances ROS control, reduces oxidative stress, and improves cardiomyocyte survival during hypoxia–reoxygenation injury.

## 2 Materials and Methods

### 2.1 Cell Culture

HL‐1 cardiomyocytes, originally established by Prof. W.C. Claycomb (CVCL_0303; LSU Health Sciences Center, New Orleans, LA, USA), were obtained from Prof. Claycomb's laboratory and from Sigma‐Aldrich (Milan Italy). Cells were maintained according to Prof. Claycomb's protocol in complete Claycomb medium supplemented with 10% fetal bovine serum (FBS; Sigma‐Aldrich, Milan, Italy), 2 mmol/L L‐glutamine (Thermo Fisher Scientific, Milan, Italy), and 100 μmol/L norepinephrine (Sigma‐Aldrich, Milan, Italy) [13]. Cells were used for no more than 10 passages. Ischaemic/hypoxic stress was induced by exposing the cells to 1% O_2_, 5% CO_2_, and 94% N_2_ in a modular incubator chamber for different times, as specified in the legends, in serum‐ and glucose‐free Dulbecco's Modified Eagle Medium (DMEM). Where indicated, ischaemic/hypoxic stress procedure was followed by 30 min of reoxygenation in normal culturing conditions. To assess the cell metabolic activity readout, cells at baseline or after ischaemic stress were incubated with 1 mL of the solution of 3‐(4,5‐Dimethylthiazol‐2‐yl)‐2,5‐diphenyltetrazole (MTT) (Sigma‐Aldrich, Milan, Italy) at a final concentration of 0.1 mg/mL in DMEM without phenol red for each well of a 12 well. After 2 h of incubation in a CO_2_ incubator at 37°C in the dark with MTT, the supernatant was aspirated and the formazan crystals were dissolved in 150 μL/well of dimethyl sulfoxide (DMSO). The absorbance per well was measured at a wavelength of 570 nm with a subtraction of background at 690 nm, using an Infinite 200 microplate reader (TECAN, Mannedorf, Switzerland).

### 2.2 PCYOX1 Overexpression in CHO Cells

CHO cells overexpressing PCYOX1 were generated as previously described [8]. Briefly, Flp‐In CHO cells (CVCL_U424; Invitrogen, Thermo Fisher Scientific, Milan, Italy) were co‐transfected with either an empty pcDNA5/FRT vector or a custom pcDNA5/FRT:PCYOX1 construct (Invitrogen, Thermo Fisher Scientific, Milan, Italy) together with the pOG44 expression vector (V6005‐20; Invitrogen, Thermo Fisher Scientific, Milan, Italy). PCYOX1‐expressing clones were then selected according to the Flp‐In System protocol (K6010‐01; Invitrogen, Thermo Fisher Scientific, Milan, Italy).

### 2.3 RNA Extraction and Real‐Time Quantitative Reverse Transcriptase PCR (qRT‐PCR)

Left ventricle from 20‐week‐old male C57/BL6J mice was homogenized using a TissueLyser II (QIAGEN, Milan, Italy). The animal procedures were approved by the Institutional Animal Care and Ethics Committee of the University of Milan and by the Ministry of Health DGSAF (N. 782‐2020). Total RNA from HL‐1 cells and homogenized tissue was extracted using the Total RNA Purification Kit (Norgen Biotek Corp., Thorold, ON, Canada), according to the manufacturer's instructions. RNA concentration and integrity were assessed using the Agilent 2100 Bioanalyzer system (Agilent Technologies, Santa Clara, California, USA). One microgram of total RNA was reverse transcribed. Real‐time qRT‐PCR was performed in triplicate using 2.5 μL of cDNA in a total reaction volume of 22.5 μL IQ Supermix containing primers and SYBRGreen fluorescence dye (Bio‐Rad Laboratories, Milan, Italy). Amplification was carried out using the iCycler Optical System (Bio‐Rad Laboratories, Milan, Italy) under the following conditions: initial denaturation at 95°C for 3 min and 30 s, followed by 50 cycles of 95°C for 15 s and 60°C for 1 min. A melting curve analysis was performed to verify amplification specificity. *Pcyox1* amplicons (120 bp) were analyzed by electrophoresis in agarose gel 2% w/v containing GelRed (Biotium, Fremont, CA, USA) and visualized with Gel doc (Bio‐Rad Laboratories, Milan, Italy). The sequences of primers used for normalization in qRT‐PCR were as follows: mouse *18s* forward: 5′‐GTA ACC CGT TGA ACC CCA TT‐3′; mouse *18s* reverse: 5′‐CCA TCC AAT CGG TAG TAG CG‐3′; mouse *Gapdh* forward: 5′‐CGT GCC GCC TGG AGA AAC C‐3′; mouse *Gapdh* reverse: 5′‐TGG AAG AGT GGG AGT TGC TGT TG‐3′. Additional gene‐specific primers were purchased from QIAGEN (Milan, Italy): mouse *Ckmt1*, QT01059254; mouse *Ckmt2*, QT00166908; mouse *Nfe2l2*, QT00095270; mouse *Ppargc1a*, QT02524242; mouse *Mybpc3*, QT00105693; mouse *Myh6*, QT00160902; mouse *Acas2l*, QT00106624; mouse *Mmp9*, QT00108815. Relative gene expression was calculated using the $2^{-∆∆C_{T}}$ method, normalizing *C*_T_ values to the housekeeping gene *18s* rRNA or *Gapdh* mRNA, as indicated.

### 2.4 Mass Spectrometry‐Based Quantification of PCYOX1

Tryptic peptides (0.5 μg/μL), obtained as described above, were combined with the stable isotope‐labeled proteotypic PCYOX1 peptide (CPSIILHD(R); Thermo Fisher Scientific, Waltham, MA, USA), used as internal standard for absolute quantification. Peptide mixtures were desalted using ZipTip C18 (Millipore, Burlington, MA, USA) according to the manufacturer's instructions, and then dissolved in 0.1% formic acid before mass spectrometry analysis. Two microliters of sample, containing 10 fmol/μL of labeled heavy peptide, were injected into a Xevo TQ‐S micro triple quadrupole mass spectrometer coupled to a Waters ACQUITY ultra‐performance liquid chromatography (UPLC) M‐Class system through an ionKey source (Waters Corporation, Milford, MA, USA), and analyzed as previously described [14, 15]. Quantification of endogenous PCYOX1 was achieved by calculating the peak area ratio between the endogenous and the heavy‐labeled peptide. The mass spectrometry proteomics data have been deposited to the ProteomeXchange Consortium via the PRIDE (SCR_003411) [16] partner repository with the dataset identifiers PXD040095 and PXD040093.

### 2.5 Enzymatic Activity Assays

PCYOX1 enzymatic activity was quantified by measuring hydrogen peroxide (H_2_O_2_) production using the Amplex Red Hydrogen Peroxide/Peroxidase Assay Kit (Life Technologies, Milan, Italy), as previously described [8, 12]. Creatine kinase (CK) activity was determined using the EnzyChrom Creatine Kinase Assay Kit (BioAssay Systems, Hayward, CA, USA), according to the manufacturer's instructions. Lactate dehydrogenase (LDH) activity was assessed by using the Lactate Dehydrogenase (LDH) Assay Kit (Abcam, Cambridge, UK) following the manufacturer's instructions.

### 2.6 Stable Transfection With Short Hairpin RNA (shRNA)

Stable silencing of *Pcyox1* was achieved using shRNA lentiviral particles (Santa Cruz Biotechnology, Dallas, Texas, USA), generated from a pool of three target‐specific plasmids encoding 19–25 nt shRNA sequences (plus hairpin) directed against *Pcyox1*. A control lentiviral construct encoding a non‐targeting scrambled shRNA sequence was used as a negative control. Cells at 50%–70% confluence were transduced for 24 h at a multiplicity of infection (MOI) of 5 in the presence of polybrene (5 μg/mL; Santa Cruz Biotechnology, Dallas, Texas, USA) in 12‐well plates. After transduction, cells were cultured in complete medium containing puromycin (2 μg/mL) to select stably transduced cells. To generate a double knock‐down cellular model silenced for *Pcyox1* and *Ckmt1* genes, stable *Pcyox1* silenced HL‐1 cells were treated with a pool of four shRNA plasmids (OriGene Technologies Inc. Rockville, USA), each encoding a 29mer target‐specific shRNA designed to knock down *Ckmt1* gene expression. A shRNA plasmid encoding for a scrambled shRNA sequence was used as a negative control. A MOI of 100 was selected to transfect cells in combination with 8 μg/mL of the transfection reagent TransduceIT (Mirus Bio LLC, Madison, USA) for each well of a 12 well/plate.

### 2.7 Apoptosis Assay

Apoptosis was assessed by quantifying cytoplasmic histone‐associated DNA fragments (mono‐ and oligonucleosomes) using a one‐step sandwich immunoassay (Cell death detection ELISA, Roche Diagnostics, Mannheim, Germany), according to the manufacturer's instructions [17]. Briefly, the amount of mono‐ and oligonucleosomes generated from the apoptotic cells was quantified using an antibody directed against histones and DNA. Data are expressed as absorbance at 405 nm (reference wavelength 490 nm) normalized to total protein content (μg).

### 2.8 Intracellular Reactive Oxygen Species Formation

Intracellular ROS generation was assessed using 2′,7′‐dichlorofluorescein diacetate (DCFH‐DA). HL‐1 cells were loaded with 10 μmol/L DCFH‐DA for 1 h at 37°C. DCFH‐DA is a lipophilic, non‐fluorescent probe that diffuses across the plasma membrane and is deacetylated to DCFH, an oxidant‐sensitive intermediate; oxidation of DCFH results in the formation of the highly fluorescent 2′,7′‐dichlorofluorescein (DCF). After incubation, cells were washed in phosphate‐buffered saline (PBS) and maintained in serum‐free, phenol‐free medium for the indicated times. Fluorescence was recorded at the indicated time points (excitation 485 nm, emission 535 nm) using an Infinite 200 microplate reader (TECAN, Mannedorf, Switzerland). Results are expressed as fluorescence units after blank subtraction (wells containing cells without DCFH‐DA) and are reported at baseline (before ischaemic/hypoxic stress) and after 3 h of ischaemic/hypoxic stress.

### 2.9 Cell Senescence Assay

Cell senescence was assessed using the Cellular Senescence Activity Assay (Enzo Life Science, Farmingdale, New York, USA), according to the manufacturer's instructions. Briefly, acidic senescence‐associated ß‐galactosidase (SA‐ß‐Gal) activity was evaluated using a fluorometric substrate. Data are reported as fluorescence units (FU).

### 2.10 Immunoassays

Tumor necrosis factor (TNF)‐α and interleukin (IL)‐6 levels were quantified using the mouse TNF‐α ELISA (Invitrogen, Milan, Italy) and mouse IL‐6 ELISA Kit (Invitrogen, Milan, Italy), respectively, according to the manufacturer's instructions.

### 2.11 Label‐Free Mass Spectrometry (LC‐MS^E^ ) Analysis

Proteomic analysis was performed on whole cells, and mitochondria homogenized in 25 mmol/L NH_4_HCO_3_ containing 0.1% RapiGest (Waters Corporation, Milford, MA, USA). Lysates were sonicated and centrifuged at 13 000 × *g* for 10 min. Fifty micrograms of protein per sample were incubated at 80°C for 15 min, reduced with 5 mmol/L dithiothreitol at 60°C for 15 min, and alkylated by carbamidomethylation with 10 mmol/L iodoacetamide for 30 min at room temperature in the dark. Sequencing‐grade trypsin (2.5 μg; Promega, Milan, Italy) was added to each sample, and digestion was carried out overnight at 37°C. Digestion was stopped by addition of 2% (v/v) trifluoroacetic acid to hydrolyze RapiGest and inactivate trypsin. Label‐free LC‐MS^E^ analysis was performed on a hybrid quadrupole‐time of flight mass spectrometer (Synapt XS; Waters Corporation, Milford, MA, USA) coupled to a UPLC M‐Class system and equipped with a nanosource (Waters Corporation, Milford, MA, USA). Samples were loaded onto a Symmetry C18 nanoACQUITY trap column, 100 Å, 5 μm, 180 μm × 2 cm (Waters Corporation, Milford, MA, USA), and separated on an HSS T3 C18 (100 Å, 1.7 μm, 75 μm × 150 mm; Waters Corporation, Milford, MA, USA) at a flow rate of 300 nL/min. Peptides were eluted using a linear gradient of solvent B from 3% to 40% over 90 min, where solvent A was 0.1% (v/v) formic acid in H_2_O and solvent B was 0.1% (v/v) formic acid in acetonitrile. Analyses were performed in biological and technical triplicates. Data were acquired in ion mobility–enhanced data‐independent acquisition mode (IMS‐DIA) using an LC–MS^E^ workflow, as previously described [18], with some modifications. The acquisition time per function was 0.5 s, with a 0.1 s inter‐scan delay. In low‐energy MS mode, data were collected at a constant collision energy of 6 eV; in high‐energy mode, fragmentation was obtained by applying drift time‐specific collision energies [19]. Progenesis QI for proteomics software (Version 4.0, http://www.nonlinear.com) was used for peptide feature quantification and protein identification.

### 2.12 Gene Ontology Analysis

Enrichment analysis of Gene Ontology (GO) terms (biological process, molecular function, cellular component) was performed using the STRING database (SCR_005223, v 10.5) [20, 21]. Enrichment *p*‐values were calculated by STRING using a hypergeometric test with Benjamini–Hochberg correction for multiple testing; terms with adjusted *p* < 0.05 were considered significant. Network construction used a maximum of five first‐shell interactors and an interaction score threshold set to medium confidence (0.4). To identify functional modules within the network, we applied Markov Cluster Algorithm (MCL) with an inflation parameter of 3, which enhances cluster granularity by increasing the stringency of network partitioning. The restriction to ≤ 5 first‐shell interactors was chosen to limit network expansion to proteins closely related to our experimentally identified set and to reduce noise from distal interactors, thereby improving interpretability of pathway‐level alterations associated with *Pcyox1* silencing.

### 2.13 Isolation of Mitochondria

Mitochondria were isolated from 2 × 10^6^ confluent HL‐1 cells using the Mitochondria Isolation Kit (Merck, Darmstadt, Germany), according to the manufacturer's instructions.

### 2.14 Mitochondrial Activity

The enzymatic activities of mitochondrial complexes I and II/III were determined in isolated mitochondria using the mitoCheck Complex I Activity Assay Kit and the mitoCheck Complex II/III Activity Assay Kit (Cayman Chemical, Ann Arbor, MI, USA), according to the manufacturer's instructions.

### 2.15 Immunoblotting

Proteins were resolved by SDS‐PAGE and transferred onto nitrocellulose membranes. After transfer, membranes were stained with MemCode reversible protein stain (Pierce Biotechnology, Cramlington, UK) to verify equal protein loading and to enable total‐protein normalization. For selected proteins (e.g., MYBPC3, MYH6), differential expression was additionally confirmed by immunoblotting using antibodies validated in HL‐1 cells. Membranes were incubated with primary antibodies against MYBPC3 (AB_2678720; Sigma‐Aldrich, Milan, Italy), MYH6 (Boster Biological Technology, Pleasanton, CA, USA), and PCYOX1 (AB_10676660; Santa Cruz Biotechnology, Dallas, Texas, USA) as previously described [8]. Densitometric data are expressed as the ratio between the band volume, after local background subtraction, and the corresponding total protein signal quantified by MemCode staining. Where a fully specific antibody was not available, protein quantification was derived exclusively from label‐free proteomic measurements, based on the detection of defined proteotypic peptides, thereby ensuring high analytical specificity.

### 2.16 Statistical Analysis

Data analysis was performed using GraphPad Prism 5.0 (SCR_002798; GraphPad Software Inc., San Diego, CA) and analyzed using the unpaired 2‐tailed Student's *t‐*test. ANOVA was performed in case of more than two conditions, with the Tukey post hoc test. Statistical significance level was considered at *p*‐value < 0.05. Data are represented as mean ± SEM.

## 3 Results

### 3.1 *Pcyox1* Silencing Protects Cardiac Muscle Cell Line HL‐1 From Apoptosis

**FIGURE 1:**
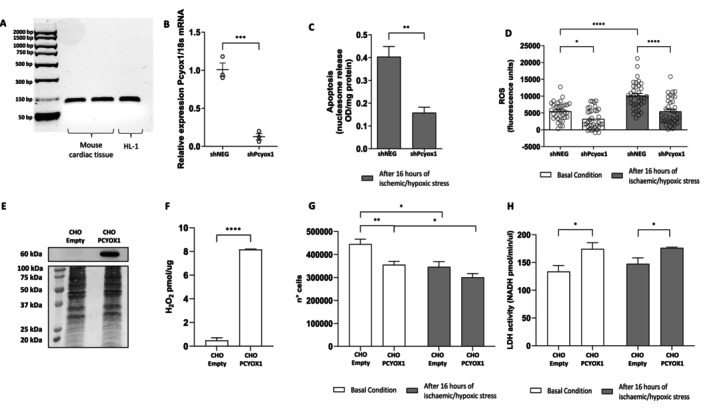
*Pcyox1* silencing reduces apoptosis and ROS production in HL‐1 cardiomyocytes wherease its overexpression reduces cell viability in *PCYOX1* overexpressing CHO cells. (A) *Pcyox1* mRNA expression in the adult mouse left ventricle cardiac tissue and HL‐1 cells assessed by qualitative RT‐PCR. (B) mRNA levels in HL‐1 cardiomyocytes stably transfected with sh*Pcyox1* lentiviral particles (shPcyox1) and in control cells treated with lentiviral particles containing a negative construct (shNEG), *n* = 3. ***p < 0.001 vs. shNEG by Student's *t*‐test. (C) Apoptosis measured by nucleosome release after 16 h of ischaemic/hypoxic stress. Data are expressed as optical density (OD) normalized to mg protein, *n* = 3. ****p < 0.0001 vs. shNEG by Student's *t*‐test. (D) ROS production measured by DCFH‐DA assay under basal conditions (white columns) and after 3 h of ischaemic/hypoxic stress (gray columns), *n* = 36. **p < 0.01, ****p < 0.0001 by ANOVA and Tukey post hoc test. (E) Blot (upper panel) and MemCode reversible protein stain (lower panel) of PCYOX1 protein in CHO control cells (CHO Empty) and CHO overexpressing PCYOX1 protein (CHO PCYOX1). (F) PCYOX1 enzimatic activity of CHO Empty and PCYOX1 expressed as pmol/μg of H_2_O_2_ produced, *n* = 3. ****p < 0.0001 vs. CHO Empty by Student's *t*‐test. (G) Cell count of Empty and PCYOX1 overexpressing CHO cells in basal conditions and after 16 h of ischaemic/hypoxic stress, *n* = 6. *p < 0.5, **p < 0.01. (H) LDH activity of CHO cells Empty and overexpressing PCYOX1 expressed as pmol/min/μl of generated NADH, *n* = 6. *p < 0.5.

We first assessed the expression of PCYOX1 in HL‐1 cardiomyocytes. As shown in Figure 1A, *Pcyox1* mRNA was detected in both HL‐1 cells and adult mouse left ventricle tissue. PCYOX1 protein was also detected in HL‐1 cells by quantitative targeted mass spectrometry (7173 ± 207.26 amol/μg protein, *n* = 3), based on the identification of a proteotypic PCYOX1 peptide. Consistent with protein expression, PCYOX1 enzymatic activity was measurable in HL‐1 lysates, generating H_2_O_2_ at a rate of 0.11 ± 0.012 pmol/μg protein (*n* = 3).

To investigate the role of PCYOX1 in ischaemic/hypoxic stress‐induced injury, we generated an HL‐1 model with stable *Pcyox1* silencing. Silencing reduced *Pcyox1* mRNA levels by 86.7% ± 4.8% (Figure 1B). Concordantly, quantitative mass spectrometry indicated that PCYOX1 protein decreased to undetectable levels in the *Pcyox1*‐silenced cells.

Because ischaemic/hypoxic stress is known to increase apoptosis in cardiomyocytes [17], we next evaluated whether *Pcyox1* silencing modulates apoptosis under ischaemic/hypoxic stress conditions. *Pcyox1* silencing significantly reduced apoptosis following ischaemic/hypoxic exposure (Figure 1C). Given the close link between oxidative stress and apoptosis [22], we quantified intracellular ROS using DCF fluorescence. As shown in Figure 1D, the lack of PCYOX1 markedly reduced ROS production both under basal conditions and during ischaemic/hypoxic stress. In control cells (shNEG), hypoxic stress increased ROS from 5497 ± 450 FU at baseline to 10 130 ± 666.7 FU (Δ = 4633 ± 762 FU). In contrast, *Pcyox1*‐silenced cells (shPcyox1) exhibited lower baseline ROS levels (3214 ± 494.6 FU) and a smaller increase under hypoxia (5455 ± 624.2 FU; Δ = 2241 ± 748.4 FU). Notably, ROS levels in hypoxic shPcyox1 cells were close to baseline values observed in control cells, indicating a blunted oxidative response in the absence of PCYOX1. Moreover, *Pcyox1*‐silenced cells not exposed to ischaemic/hypoxic stress exhibited lower basal ROS compared with control cells, suggesting that PCYOX1 also contributes to ROS generation under basal conditions (Figure 1D). Since premature senescence may occur under ischaemic/hypoxic conditions [23], we assessed senescence‐associated β‐galactosidase activity. *Pcyox1* silencing did not significantly affect senescence (29 830 ± 1310 FU and 31 709 ± 1661 FU in shNEG cells and shPcyox1 cells, respectively, *p* = 0.311, *n* = 3). In response to ischaemic/hypoxic conditions, cytokine production may increase [24], and MMP‐9 has been implicated as a critical mediator of cardiac dysfunction [25]. *Pcyox1* silencing did not alter IL‐6 and TNFα levels, nor *Mmp9* expression, under basal conditions and after ischaemic/hypoxic stress (Figure S1).

To complement the loss‐of‐function approach in HL‐1 cardiomyocytes, we next evaluated the effects of PCYOX1 overexpression in Flp‐In CHO cells. Stable overexpression resulted in a marked increase in PCYOX1 protein levels, as confirmed by immunoblotting and quantitative analysis, and in a significant enhancement of PCYOX1 enzymatic activity, measured as H_2_O_2_ production (Figure 1E,F). Functionally, PCYOX1‐overexpressing CHO cells exhibited a significant reduction in cell number under basal conditions compared with control‐transfected cells (Figure 1G). A similar trend was observed after exposure to hypoxic conditions, although the difference did not reach statistical significance. In parallel, LDH release, used as an index of cytotoxicity, was increased in PCYOX1‐overexpressing cells both at baseline and following hypoxic stress (Figure 1H).

### 3.2 Pcyox1 Silencing Preserves the Cardiomyocytes' Proteome Under Ischaemic/Hypoxic Stress Conditions

**FIGURE 2:**
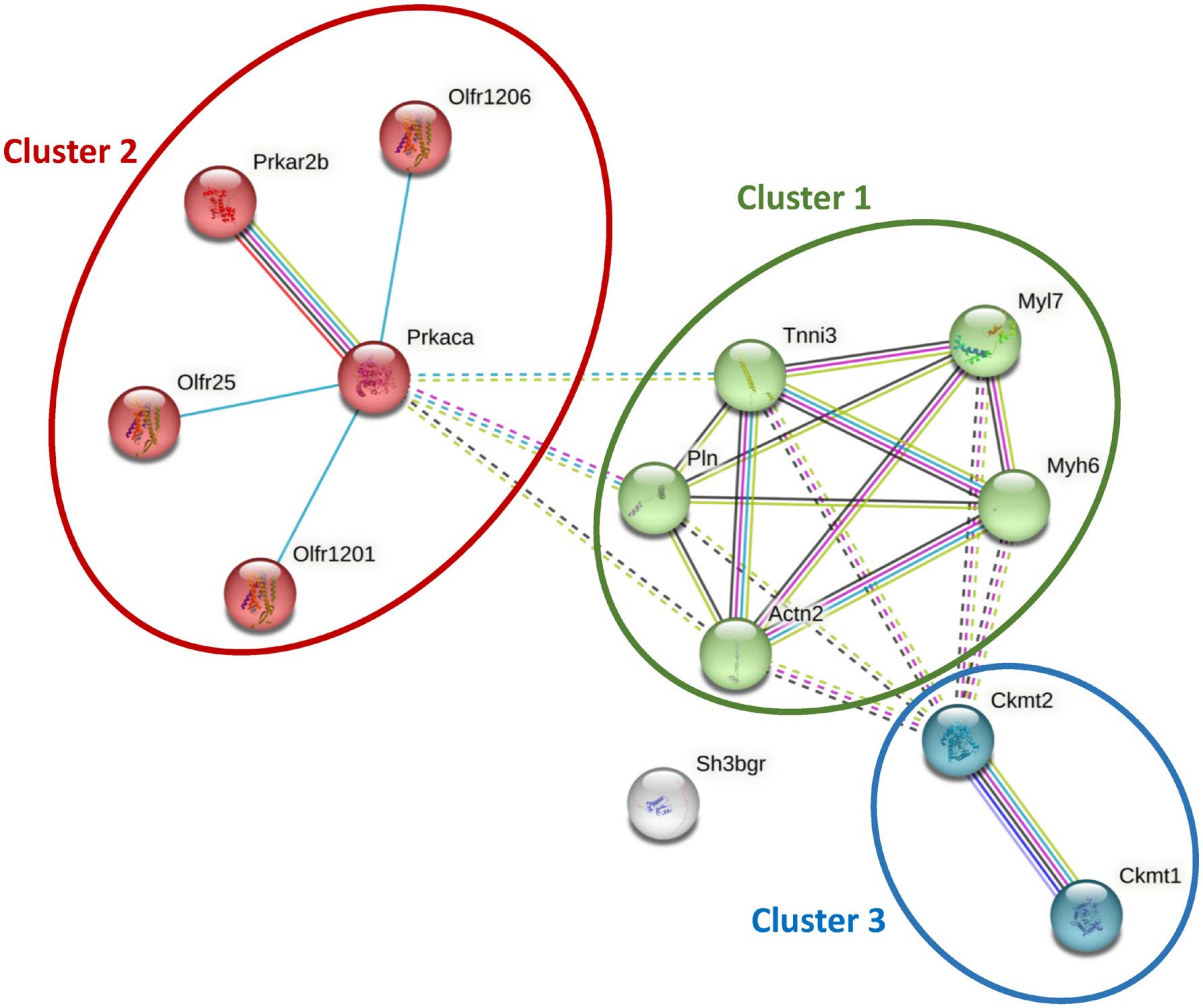
STRING‐based protein–protein interaction (PPI) network and functional enrichment analysis of proteins upregulated in *Pcyox1*‐silenced cells. Green nodes (Cluster 1) represent proteins associated with striated muscle contraction; red nodes (Cluster 2) correspond to proteins involved in PKA activation and cAMP signaling; blue nodes (Cluster 3) indicate proteins related to phosphocreatine biosynthesis. Solid edges represent high‐confidence interactions supported by curated databases or experimental evidence, whereas dashed edges denote predicted associations based on gene co‐occurrence, co‐expression, or text mining.

*Pcyox1* silencing significantly reshaped the cellular proteome under basal conditions (Table 1). Specifically, eight proteins were increased in *Pcyox1*‐silenced cells, with functional enrichment pointing to pathways related to CK activity (*p* = 0.0078, *n* = 2), cAMP‐dependent protein kinase complex (*p* = 0.0088, *n* = 2), and striated muscle contraction (*p* = 0.00042, *n* = 3). This proteomic signature was largely maintained during ischaemic/hypoxic stress conditions (Table S1) and after reoxygenation (Table S2). Notably, CK, cAMP‐dependent protein kinase catalytic subunit alpha, myosin‐6, and myosin regulatory light chain 2 atrial isoform remained consistently elevated in *Pcyox1*‐silenced cells after ischaemic/hypoxic stress conditions and reoxygenation (Tables S1 and S2). Figure 2 shows the STRING‐derived protein–protein interaction (PPI) network of proteins upregulated in *Pcyox1*‐silenced cells, with cluster analysis highlighting three functionally distinct modules. Cluster 1 grouped 5 proteins (alpha‐actinin‐2, Actn2; troponin I, cardiac muscle, Tnni3; phospholamban, Pln; myosin regulatory light chain 2, atrial isoform, Myl7; and myosin‐6, Myh6) associated with striated muscle contraction, suggesting relevance for cardiomyocyte structural integrity and contractile function. Cluster 2 comprised 5 proteins (cAMP‐dependent protein kinase catalytic subunit alpha, Prkaca; olfactory receptor, Olfr1201; olfactory receptor, Olfr25; olfactory receptor, Olfr1206; and cAMP‐dependent protein kinase type II‐beta regulatory subunit, Prkarb2) primarily linked to PKA activation and cAMP‐mediated signaling. Cluster 3 consisted of 2 proteins (creatine kinase U‐type mitochondrial, Ckmt1 and creatine kinase S‐type mitochondrial, Ckmt2) involved in phosphocreatine biosynthesis, implicating a role in cellular energy buffering and mitochondrial metabolism. These clusters form biologically coherent groupings that are consistent with the known functions of the constituent proteins and support the interpretation of PCYOX1‐dependent proteomic remodeling under hypoxic stress.

**TABLE 1:** List of proteins modulated by silencing of *Pcyox1* in HL‐1 under basal conditions.

Accession	Unique peptides	Score	Anova (*p*)	Max fold change	Description
Proteins increased with *Pcyox1* silencing					
**Q6P8J7**	**5**	**58.0167**	**3.82E‐06**	**2.64**	**Creatine kinase S‐type_mitochondrial OS = *Mus musculus* GN = Ckmt2 PE = 1 SV = 1**
P31324	5	60.6699	0.003974	**2.41**	cAMP‐dependent protein kinase type II‐beta regulatory subunit OS = *Mus musculus* GN = Prkar2b PE = 1 SV = 3
**Q02566**	**21**	**1040.69**	**2.53E‐07**	**2.03**	**Myosin‐6 OS = *Mus musculus* GN = Myh6 PE = 1 SV = 2**
**P05132**	**4**	**56.1834**	**0.000438**	**1.78**	**cAMP‐dependent protein kinase catalytic subunit alpha OS = *Mus musculus* GN = Prkaca PE = 1 SV = 3**
**Q9QVP4**	**8**	**135.1404**	**0.000306**	**1.70**	**Myosin regulatory light chain 2_atrial isoform OS = *Mus musculus* GN = Myl7 PE = 1 SV = 1**
**P30275**	**5**	**65.8573**	**2.56E‐06**	**1.61**	**Creatine kinase U‐type_mitochondrial OS = *Mus musculus* GN = Ckmt1 PE = 1 SV = 1**
Q9WUZ7	4	64.2587	0.025454	**1.45**	SH3 domain‐binding glutamic acid‐rich protein OS = *Mus musculus* GN = Sh3bgr PE = 1 SV = 1
**Q9JI91**	**9**	**229.1857**	**5.45E‐06**	**1.35**	**Alpha‐actinin‐2 OS = *Mus musculus* GN = Actn2 PE = 1 SV = 2**
Proteins decreased with *Pcyox1* silencing					
**P50543**	**2**	**13.5796**	**5.93E‐05**	**3.27**	**Protein S100‐A11 OS = *Mus musculus* GN = S100a11 PE = 1 SV = 1**
**Q9Z0P4**	**2**	**27.6714**	**0.016985**	**2.28**	**Paralemmin‐1 OS = *Mus musculus* GN = Palm PE = 1 SV = 1**
Q02248	3	57.5165	0.002519	**2.08**	Catenin beta‐1 OS = *Mus musculus* GN = Ctnnb1 PE = 1 SV = 1
**Q64337**	**9**	**60.3814**	**0.003095**	**1.81**	**Sequestosome‐1 OS = *Mus musculus* GN = Sqstm1 PE = 1 SV = 1**
**O88792**	**3**	**60.6055**	**0.001479**	**1.69**	**Junctional adhesion molecule A OS = *Mus musculus* GN = F11r PE = 1 SV = 2**
P17225	7	130.6884	0.003644	**1.46**	Polypyrimidine tract‐binding protein 1 OS = *Mus musculus* GN = Ptbp1 PE = 1 SV = 2
P52293	7	69.0227	0.002433	**1.44**	Importin subunit alpha‐1 OS = *Mus musculus* GN = Kpna2 PE = 1 SV = 2
Q6IRU2	3	56.5043	7.57E‐05	**1.34**	Tropomyosin alpha‐4 chain OS = *Mus musculus* GN = Tpm4 PE = 1 SV = 3

*Note:* Proteins modulated in the same way in the proteome of cells under basal conditions, after ischaemic/hypoxic stress, and after reoxygenation are in bold (see also Tables S1 and S2, respectively).

Conversely, *Pcyox1* silencing reduced the abundance of several proteins under basal conditions including S100‐A11 (Calcium Binding Protein A11), Paralemmin 1, and Catenin beta‐1 (Table 1). Importantly, a subset of the downregulated proteins, such as S100‐A11, Paralemmin 1, Junctional adhesion molecule A, and Sequestosome‐1, was similarly decreased during ischaemic/hypoxic stress and after reoxygenation (Tables S1 and S2) indicating that *Pcyox1* silencing induces a conserved proteomic shift across experimental conditions.

**FIGURE 3:**
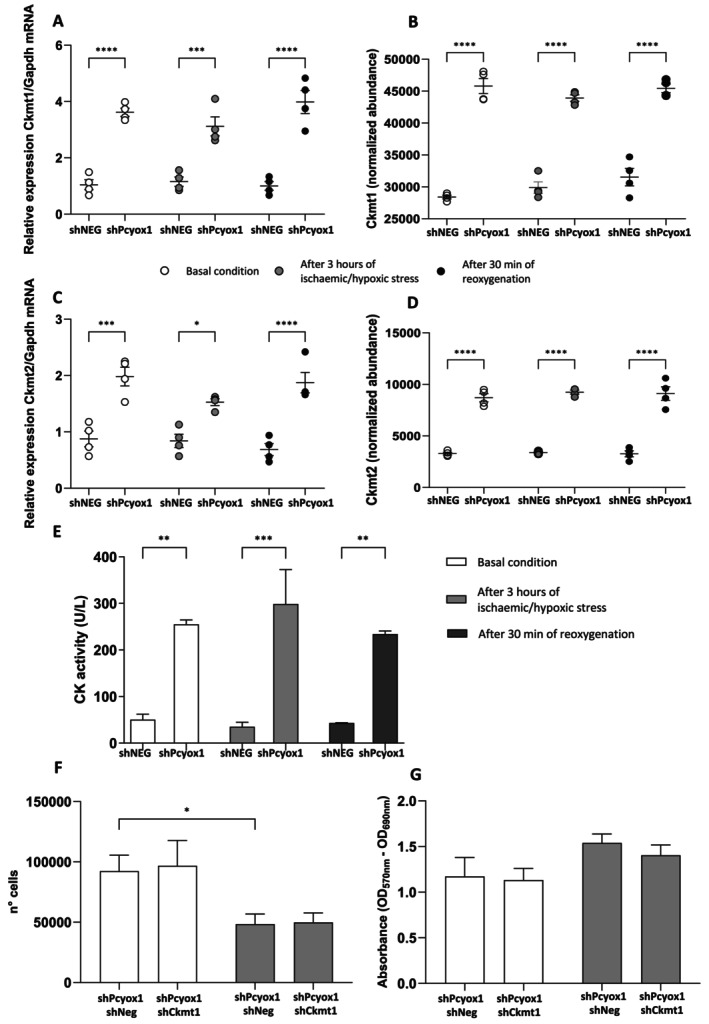
*Pcyox1* silencing modulates the creatine kinase (CK) system in HL‐1 cells. (A, B) *Ckmt1* mRNA (A) and CKMT1 protein abundance (B) in shNEG and shPcyox1 cells under the indicated experimental conditions (*n* = 4). (C, D) *Ckmt2* mRNA (C) and CKMT2 protein abundance (D) in shNEG and shPcyox1 cells under the indicated experimental conditions (*n* = 4). (E) Total CK activity in shNEG and shPcyox1 cells at baseline (white bars), after 3 h of ischaemic/hypoxic stress (gray bars), and after 30 min of reoxygenation (black bars) (*n* = 3–4). (F, G) Rescue experiments. HL‐1 cells stably silenced for *Pcyox1* were transduced with either control shRNA (shNEG) or shCkmt1. (F) Cell number determined by direct counting and (G) MTT assay (expressed as absorbance) under basal conditions (white bars) and after 16 h of ischaemic/hypoxic stress (gray bars) (*n* = 3–4). Statistical analysis: ANOVA followed by Tukey's post hoc test. *p < 0.05; **p < 0.01; ***p < 0.001; ****p < 0.0001.

To further support the quantitative proteomic findings, we assessed the levels of CK mRNA (Figure 3A,C). *Pcyox1* silencing significantly increased *Ckmt1* and *Ckmt2* mRNA, and this induction was observed under basal conditions, after ischaemic/hypoxic stress, and after reoxygenation. Consistently, label‐free quantitative proteomics by LC‐MS^E^ revealed higher Ckmt1 and Ckmt2 protein abundance across all experimental conditions (Figure 3B,D). The increase in CK expression was paralleled by enhanced enzymatic activity. As shown in Figure 3E, CK activity was significantly higher in *Pcyox1‐*silenced cells at baseline, after experiencing ischaemic/hypoxic stress, and following reoxygenation. To determine whether the upregulation of *Ckmt1* is functionally required for the cytoprotective phenotype induced by *Pcyox1* silencing, we performed double knockdown experiments by silencing *Ckmt1* in *Pcyox1*‐deficient HL‐1 cells. Under basal conditions, concomitant *Ckmt1* knockdown did not significantly alter cell number or the metabolic activity readout (MTT assay) compared with shPcyox1 controls (Figure 3F,G). Importantly, under ischaemic/hypoxic stress, the improved cell viability observed in shPcyox1 cells was preserved despite *Ckmt1* knockdown, as assessed by both direct cell counting and the metabolic activity assay (Figure 3F,G). The concordant results obtained with these two independent readouts argue against a major role for *Ckmt1* in mediating the survival advantage conferred by *Pcyox1* silencing.

### 3.3 Silencing Pcyox1 Preserves the Proteome of the Cardiac Mitochondria

Proteomic analysis revealed that *Pcyox1* silencing was associated with increased levels of both Ckmt1 and Ckmt2 at both mRNA and protein levels (Figure 3A–D). Given that mitochondria represent primary targets and amplifiers of cellular stress during hypoxia–reoxygenation [26], we next evaluated whether *Pcyox1* deficiency affected the mitochondrial proteome and the activity of respiratory chain complexes. Mitochondria were isolated using a standardized method developed within the framework of the “The Mitochondrial Human Proteome Project” (mt‐HPP), ensuring high purity and integrity of the mitochondrial fraction for subsequent functional assays and proteomic analysis [27]. PCYOX1 was detected in the mitochondrial fraction, and its silencing resulted in significant remodeling of the mitochondrial proteome (Table 2) including enrichment of Ckmt1 and Ckmt2.

**TABLE 2:** List of mitochondrial proteins modulated by silencing of *Pcyox1* in HL‐1 cells.

Accession	Unique peptides	Score	Anova (*p*)	Max fold change	Description
Mitochondrial proteins increased with *Pcyox1* silencing					
Q99NB1	2	17.7	1.9E‐03	9.1	Acetyl‐coenzyme A synthetase 2‐like_mitochondrial OS = *Mus musculus* GN = Acss1
P09542	4	54.9	2.6E‐09	7.64	Myosin light chain 3 OS = *Mus musculus* GN = Myl3
Q9Z2H5	2	17.8	3.5E‐04	6.5	Band 4.1‐like protein 1 OS = *Mus musculus* GN = Epb41l1
P49586	2	18.2	1.4E‐04	2.67	Choline‐phosphate cytidylyltransferase A OS = *Mus musculus* GN = Pcyt1a
P09541	9	157.5	5.5E‐07	2.5	Myosin light chain 4 OS = *Mus musculus* GN = Myl4
Q02566	38	1668.6	4.0E‐07	2.44	Myosin‐6 OS = *Mus musculus* GN = Myh6
Q9QVP4	13	181.6	2.3E‐06	2.28	Myosin regulatory light chain 2_atrial isoform OS = *Mus musculus* GN = Myl7
Q9D6U8	7	104.0	7.5E‐03	2.18	Protein FAM162A OS = *Mus musculus* GN = Fam162a
O70468	11	81.4	4.8E‐02	2.13	Myosin‐binding protein C_cardiac‐type OS = *Mus musculus* GN = Mybpc3
P30275	9	107.1	1.1E‐07	1.97	Creatine kinase U‐type_mitochondrial OS = *Mus musculus* GN = Ckmt1
P82348	2	12.9	2.9E‐05	1.93	Gamma‐sarcoglycan OS = *Mus musculus* GN = Sgcg
P31324	3	16.1	2.1E‐04	1.89	cAMP‐dependent protein kinase type II‐beta regulatory subunit OS = *Mus musculus* GN = Prkar2b
Q9CPU4	2	24.6	4.5E‐02	1.88	Microsomal glutathione S‐transferase 3 OS = *Mus musculus* GN = Mgst3
P48542	2	30.9	2.9E‐04	1.84	G protein‐activated inward rectifier potassium channel 2 OS = *Mus musculus* GN = Kcnj6
Q6P8J7	6	88.3	1.6E‐03	1.78	Creatine kinase S‐type_mitochondrial OS = *Mus musculus* GN = Ckmt2
P97447	4	44.1	1.9E‐06	1.7	Four and a half LIM domains protein 1 OS = *Mus musculus* GN = Fhl1
P50462	8	94.9	5.4E‐05	1.55	Cysteine and glycine‐rich protein 3 OS = *Mus musculus* GN = Csrp3
Q9CZS1	20	245.2	8.7E‐03	1.53	Aldehyde dehydrogenase X mitochondrial OS = *Mus musculus* GN = Aldh1b1
Mitochondrial proteins decreased with Pcyox silencing					
Q9R1Q6	2	14.2	1.5E‐07	3.76	Transmembrane protein 176B OS = *Mus musculus* GN = Tmem176b
Q64737	2	11.4	1.5E‐05	2.97	Trifunctional purine biosynthetic protein adenosine‐3 OS = *Mus musculus* GN = Gart
Q8R0W6	2	12.0	6.7E‐08	2.61	NEDD4 family‐interacting protein 1 OS = *Mus musculus* GN = Ndfip1
Q06335	2	10.3	2.1E‐04	2.06	Amyloid‐like protein 2 OS = *Mus musculus* GN = Aplp2
Q62371	2	16.6	2.1E‐04	1.95	Discoidin domain‐containing receptor 2 OS = *Mus musculus* GN = Ddr2
Q9JHP7	2	18.8	1.0E‐05	1.89	KDEL motif‐containing protein 1 OS = *Mus musculus* GN = Kdelc1
Q9CQF9	5	47.7	2.5E‐05	1.87	Prenylcysteine oxidase OS = *Mus musculus* GN = Pcyox1
Q60961	4	46.1	1.3E‐06	1.76	Lysosomal‐associated transmembrane protein 4A OS = *Mus musculus* GN = Laptm4a
Q91VK4	2	20.3	3.4E‐02	1.73	Integral membrane protein 2C OS = *Mus musculus* GN = Itm2c
P97370	3	21.0	1.3E‐02	1.6	Sodium/potassium‐transporting ATPase subunit beta‐3 OS = *Mus musculus* GN = Atp1b3
Q9DBE8	4	25.8	7.9E‐08	1.6	Alpha‐1_3/1_6‐mannosyltransferase ALG2 OS = *Mus musculus* GN = Alg2
P47738	18	198.4	1.7E‐04	1.59	Aldehyde dehydrogenase_mitochondrial OS = *Mus musculus* GN = Aldh2
Q09143	3	16.7	4.0E‐03	1.58	High affinity cationic amino acid transporter 1 OS = *Mus musculus* GN = Slc7a1
O55028	4	22.6	2.2E‐06	1.57	[3‐methyl‐2‐oxobutanoate dehydrogenase [lipoamide]] kinase_mitochondrial OS = *Mus musculus* GN = Bckdk
P28656	3	34.1	1.5E‐02	1.53	Nucleosome assembly protein 1‐like 1 OS = *Mus musculus* GN = Nap1l1
Q9D8Y0	2	12.2	2.7E‐04	1.52	EF‐hand domain‐containing protein D2 OS = *Mus musculus* GN = Efhd2
Q499X9	3	18.4	2.5E‐03	1.51	Methionine‐tRNA ligase_mitochondrial OS = *Mus musculus* GN = Mars

**FIGURE 4:**
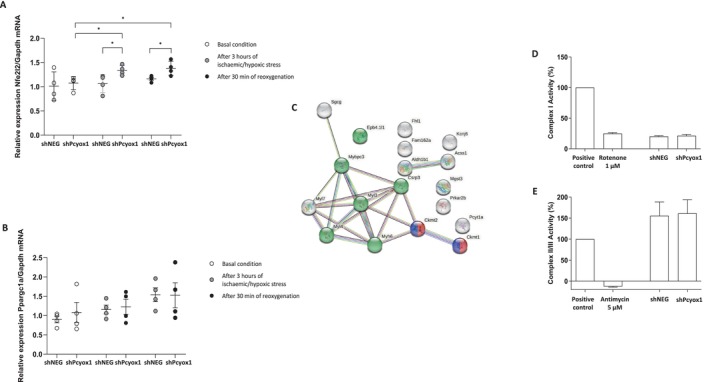
Effects of *Pcyox1* silencing on the mitochondrial proteome of HL‐1 cells and on the activity of mitochondrial respiratory chain complex I and complex II/III. (A, B) mRNA levels of *Nfe2l2* (A) and *Ppargc1a* (B) in shNEG and shPcyox1 HL‐1 cells under basal conditions (white dots), after 3 h of ischaemic/hypoxic stress (gray dots), and after 30 min of reoxygenation (black dots), *n* = 4. *p < 0.05 by Student's *t*‐test. (C) GO enrichment analysis of mitochondrial proteins more abundant in *Pcyox1*‐silenced cells: Red and blue nodes indicate proteins annotated to CK activity; green nodes indicate actin binding. (D) Complex I activity and (E) complex II/III activity in shNEG cells and shPcyox1 cells. Bovine mitochondrial extract was used as positive control; rotenone and antimycin were used as inhibitors of complex I and complex II/III activity, respectively. *n* = 3.

To explore whether these proteomic changes might be associated with redox‐sensitive transcriptional mechanisms, we assessed the expression of *Nfe2l2* (Nrf2) and *Ppargc1a* (PGC‐1α), two regulators of mitochondrial metabolism and oxidative stress responses [28]. *Pcyox1* silencing significantly increased *Nfe2l2* expression after ischaemic/hypoxic stress and following reoxygenation (Figure 4A). In contrast, *Ppargc1a* showed only a non‐significant upward trend under the same conditions (Figure 4B). These findings suggest that the mitochondrial adaptations observed in *Pcyox1*‐deficient HL‐1 cells may be linked, at least in part, to enhanced Nrf2‐associated transcriptional responses, whereas PGC‐1α does not appear to play a major role under the experimental conditions tested. GO analysis revealed enrichment in proteins involved in CK activity (*p* = 0.021) and actin‐binding (*p* = 0.0024) (Figure 4C). Notably, despite the observed remodeling of the mitochondrial proteome, the activity of respiratory chain complex I and complex II/III was not significantly affected (Figure 4D,E).

**FIGURE 5:**
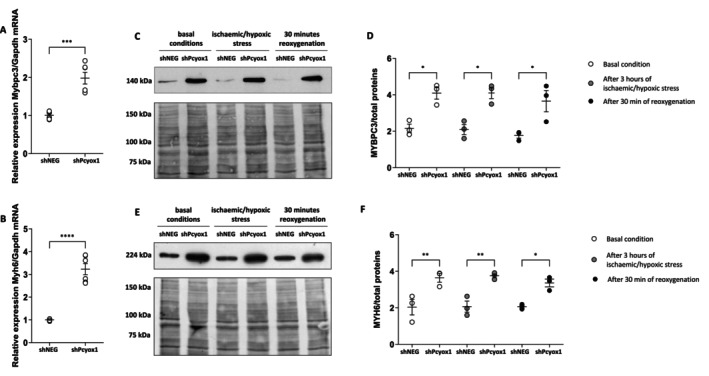
Effect of *Pcyox1* silencing on the myosin component in HL‐1 cells. mRNA levels of (A) *Mybpc3*, and (B) *Myh6* in shNEG and shPcyox1 cells in basal conditions. (C, D) Myosin‐binding protein C (MYBPC3) and (E, F) myosin‐6 (MYH6) protein levels were detected by Western blot analysis in shNEG and in shPcyox1 cells at basal conditions (white round symbols), after 3 h ischaemic/hypoxic stress (gray round symbols), and after 30 min reoxygenation (black round symbols). (C, E) Blots (upper panel) and MemCode reversible protein stain (lower panel) of myosin‐binding protein C and myosin‐6, respectively. (D, F) Densitometric analyses. Blots are representative of *n* = 3 independent experiments. *p < 0.05, **p < 0.01, ***p < 0.001, ****p < 0.0001 by ANOVA and Tukey post hoc test.

The proteomic analysis of both the mitochondrial fraction and the whole‐cell lysate indicated that several contractile and sarcomeric components, including multiple myosin‐related proteins, were more abundant in *Pcyox1*‐silenced cells than in control cells. Consistently, mRNA analysis showed that *Mybpc3* and *Myh6* transcript levels were increased following *Pcyox1* downregulation (Figure 5A,B), supporting enhanced biosynthesis of myosin‐binding protein C (MYBPC3) and myosin heavy chain 6 (MYH6; α‐myosin heavy chain, α‐MHC).

At the protein level, MYBPC3 and MYH6 abundance were also significantly higher in *Pcyox1*‐silenced cells after ischaemic/hypoxic stress and after reoxygenation (Figure 5C–F). Collectively, these data indicate that *Pcyox1* silencing is associated with increased expression of key sarcomeric proteins both at baseline and in response to hypoxia–reoxygenation challenge.

**FIGURE 6:**
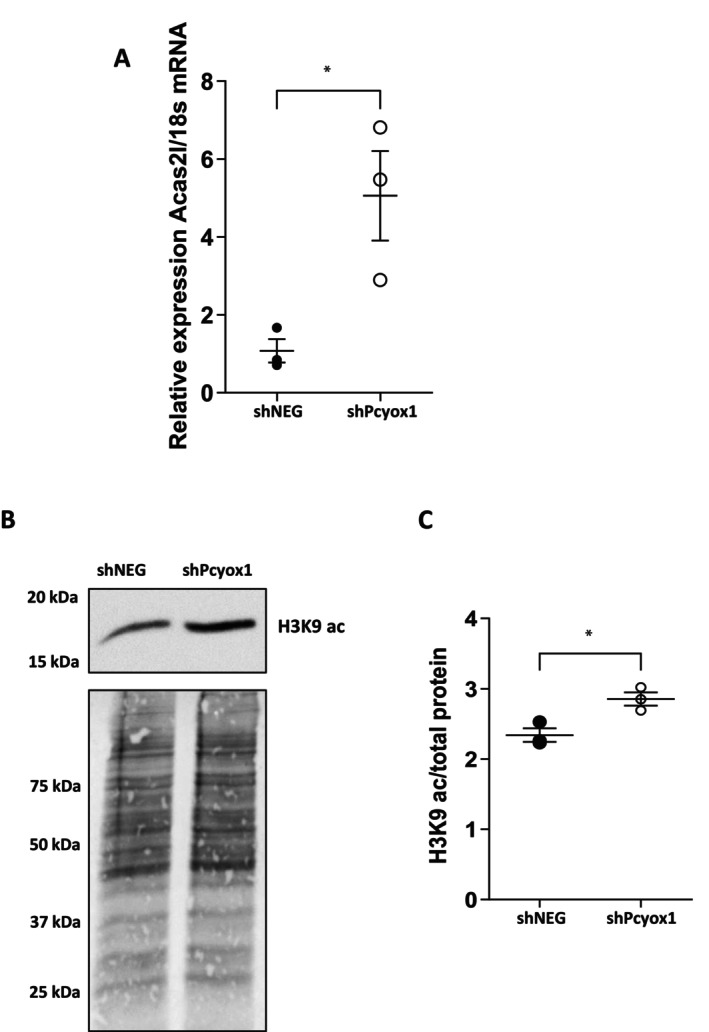
Effect of *Pcyox1* silencing on *Acsas2l* and H3K9 acetylated levels. (A) mRNA levels of *Acas2l* in shNEG and shPcyox1 cells. *18s* mRNA was used as housekeeping gene, *n* = 3. *p < 0.5 with Student's *t*‐test. (B) Representative immunoblot of histone H3 acetylated on lysine 9 (H3K9ac) in shNEG and shPcyox1 HL‐1 cells (upper panel) and MemCode reversible protein stain (lower panel). (C) Densitometric analysis of H3K9ac normalized to total protein (MemCode), *n* = 3. *p < 0.05 with Student's *t*‐test.

Moreover, proteomic analysis of the mitochondrial fraction identified acetyl‐CoA synthetase 2‐like as one of the proteins most enriched in *Pcyox1*‐silenced cells. Acetyl‐CoA synthetase 2‐like belongs to the acetyl‐Coenzyme A synthetase (ACSS) family, which contributes to acetyl‐CoA generation from acetate and thereby links acetate utilization to cellular energetic and biosynthetic pathways [29]. Consistently, mRNA analysis confirmed increased *Acas2l* mRNA levels in *Pcyox1*‐deficient cells (Figure 6A). Beyond its metabolic role, the ACSS family has been implicated in the regulation of acetylation‐dependent processes, including histone acetylation, by modulating acetyl‐CoA availability [30]. In line with this framework, *Pcyox1* silencing significantly increased histone H3 acetylation on lysine 9 (H3K9ac) (Figure 6B,C), supporting the notion that reduced PCYOX1 expression is associated with enhanced histone acetylation and suggesting a potential link between PCYOX1‐dependent redox signaling and acetyl‐CoA–driven epigenetic regulation (Figure 6B,C).

## 4 Discussion

In this study, we identify PCYOX1 as a previously underappreciated determinant of cardiomyocyte vulnerability during a hypoxia–reoxygenation insult. In HL‐1 cells, PCYOX1 ablation promoted cell survival, concomitantly reducing ROS accumulation and apoptosis, and was associated with coordinated proteomic remodeling in pathways plausibly relevant to stress tolerance both at baseline and during hypoxia–reoxygenation.

The present study was conducted using HL‐1 cardiomyocytes, an immortalized murine atrial cell line that preserves several key structural and functional properties of adult cardiomyocytes including spontaneous contractile activity, expression of cardiac‐specific contractile proteins, responsiveness to β‐adrenergic stimulation, and mitochondria‐dependent oxidative metabolism [31]. Consistent with its extensive use as an in vitro platform to interrogate mechanisms of ischaemia–reperfusion injury [32], HL‐1 cells provide a robust and reproducible system for mechanistic dissection. We nonetheless acknowledge that HL‐1 cells cannot fully recapitulate the multicellular architecture and metabolic heterogeneity of native myocardium. Accordingly, future studies in human iPSC‐derived cardiomyocytes and in vivo models will be required to establish the translational relevance of PCYOX1 modulation across species and tissue contexts.

PCYOX1 is a FAD‐dependent thioether oxidase that catalyzes the oxidative cleavage of prenylcysteines, producing free cysteine, an isoprenoid aldehyde, and a stoichiometric amount of H_2_O_2_ [33]. The biochemical mechanism proposed by Casey's group for PCYOX1 is unique in biology and distinguishes it from other enzymes that act on prenylcysteines such as flavin‐dependent monooxygenases (FMOs) or P450s, or enzymes that cleave these substrates via cysteine β‐lyases [33, 34, 35, 36].

Cardiomyocytes harbor multiple sources of ROS including NOX enzymes [37], xanthine oxidoreductase [38], and nitric oxide synthase [33]. Furthermore, at least ten sites of mitochondrial ROS production have been identified including the electron transport chain (complex I, complex II, complex III) and other enzymatic sources such as 2‐oxoglutarate dehydrogenase, pyruvate dehydrogenase, branched‐chain 2‐oxoacid dehydrogenase, mitochondrial glycerol phosphate dehydrogenase, ETF (electron‐transferring‐flavoprotein) dehydrogenase, and dihydroorotate dehydrogenase [39, 40]. In addition, monoamine oxidases A and B located at the outer mitochondrial membrane represent relevant pro‐oxidant systems in the heart [41, 42].

Against this background, the reduction of ROS observed upon *Pcyox1* silencing supports a functional contribution of PCYOX1 to cardiomyocyte redox homeostasis, particularly under ischaemic/hypoxic stress conditions. Quantitatively, ROS levels under ischaemic/hypoxic stress were 10 130 ± 666.7 FU in control cells and 5455 ± 624.2 FU in *Pcyox1*‐silenced cells, corresponding to an ~50% reduction; under hypoxic conditions, ROS levels in *Pcyox1*‐deficient cells remained close to baseline values observed in control cells. This pattern is consistent with a blunted oxidative stress response in the absence of PCYOX1. Although the involvement of additional ROS‐generating systems cannot be excluded, their net contribution appears functionally constrained in this setting, as oxidative stress and apoptosis were both markedly reduced. Collectively, these observations support the concept that PCYOX1 may act as an amplifier of ROS accumulation during hypoxia–reoxygenation rather than merely adding a marginal ROS source. The gain‐of‐function experiments performed in CHO cells further strengthen this interpretation. Stable PCYOX1 overexpression increased enzymatic activity and was associated with reduced cell number and enhanced LDH release under basal conditions, indicating increased cytotoxicity. Although the reduction in cell number under hypoxic conditions did not reach statistical significance, the consistent increase in LDH release supports a detrimental effect of excessive PCYOX1 activity on cellular integrity. Although these experiments were conducted in a non‐cardiac cellular context and therefore do not directly model cardiomyocyte‐specific responses, they provide complementary evidence that PCYOX1 overactivity is sufficient to promote cellular stress and compromise viability. Together with the protective phenotype observed upon *Pcyox1* silencing in HL‐1 cells, these findings support a model in which PCYOX1 functions as a pro‐oxidant stress amplifier whose activity level critically influences cell fate.

To explore adaptive programs associated with PCYOX1 loss, we performed quantitative proteomics in whole‐cell lysates and in mitochondrial fractions. These analyses revealed coordinated changes in proteins involved in CK activity, cAMP/PKA signaling, and striated muscle contraction, suggesting that *Pcyox1* silencing is coupled to broader metabolic and structural remodeling. The mechanisms linking reduced PCYOX1 activity to these pathways are likely multifactorial and may extend beyond ROS generation per se. With respect to mitochondrial remodeling, our data indicate that *Pcyox1* silencing increased both transcript and protein levels of mitochondrial CK (Ckmt1 and Ckmt2), compatible with a transcriptional component. We also observed increased *Nfe2l2* mRNA following ischaemic/hypoxic stress and reoxygenation, whereas *Ppargc1a* showed only a modest, non‐significant trend. Given the established role of Nrf2 in regulating metabolic and mitochondrial gene networks [28], these findings are consistent with the possibility that altered redox signaling in the absence of PCYOX1 engages Nrf2‐associated adaptive responses. However, because Nrf2 activation is classically established through functional readouts (e.g., nuclear accumulation and induction of canonical target genes), the present dataset supports an association with Nrf2‐linked transcriptional regulation rather than a definitive causal chain. Importantly, additional layers of regulation—such as mitochondrial import, selective turnover, or mitophagy—cannot be excluded, and our data do not directly address these mechanisms. Finally, exploration of protein–protein interaction resources (e.g., BioGRID, https://thebiogrid.org/119547/summary/homo‐sapiens/pcyox1.html) may help generate hypotheses on PCYOX1 functional partnerships and domain‐mediated interactions that could integrate redox signaling with broader cellular pathways.

The CK system represents a plausible functional node within this remodeling. CK isoenzymes are central to cardiac energy balance [43] and energy phosphotransfer [44]. In vertebrates, sarcomeric MtCK predominates in striated muscle, whereas ubiquitous MtCK is expressed broadly in other tissues [45]. In our model, *Pcyox1* silencing increased total CK activity under basal conditions and after exposure to ischaemic/hypoxic stress and reoxygenation. Because total CK activity was measured, increased activity may reflect contributions from cytosolic CK isoforms in addition to mitochondrial CKs. However, all our findings indicate that silencing *Pcyox1* leads to a protective effect against ischaemic/hypoxic stress and reoxygenation injury, which may be partially attributed to the increased CK activity. In line with this, recent studies have demonstrated that increased CK activity protects the HL‐1 cell line against ischaemia‐reperfusion injury [46]. Although these changes correlate with improved survival, rescue experiments showed that concomitant knockdown of *Ckmt1* in *Pcyox1*‐deficient cells did not abolish the survival advantage. Thus, *Ckmt1* upregulation is unlikely to be a single necessary driver of cytoprotection; instead, it more plausibly represents a component of a broader metabolic adaptation accompanying PCYOX1 loss, consistent with the multi‐pathway proteomic remodeling observed.

*Pcyox1* silencing also increased the levels of mitochondrial acetyl‐CoA synthetase 2 (Acss1; also annotated as acetyl‐coenzyme A synthetase 2‐like, *Acas2l*), an important member of the ACSS family. Acss1 catalyzes the conversion of acetate to acetyl‐CoA [47], an intermediate metabolite that serves as an energy‐providing substrate. In mammalian cells, ACSS enzymes and citrate‐dependent ATP‐citrate lyase (ACL) contribute to acetyl‐CoA production that can support histone acetylation [48]. Consistent with this framework, cells with silenced *Pcyox1* displayed increased H3K9ac. However, the present results do not establish whether increased H3K9ac is mechanistically mediated by Acss1 induction or reflects a broader rewiring of acetyl‐CoA metabolism and redox‐dependent epigenetic regulation.

Proteomic clustering further suggested enrichment in modules related to PKA signaling, contractile machinery, and energy metabolism, aligning with the phenotypic observations of reduced ROS accumulation and preserved cardiomyocyte viability. Notably, myosin and myosin‐associated proteins were enriched following *Pcyox1* silencing. Myosins are proteins that play critical roles in various cell functions such as force production, cell motility, cytokinesis, transportation, and organelle localization through association with actin filaments. In particular, regulatory light chains have emerged as important determinants of cardiac muscle function, modulating myosin head positioning and cross‐bridge formation during striated muscle contraction [49].

Consistent with the link between contractile protein integrity and survival, Communal et al. [50] reported that cleavage of myosin heavy chain and myosin light chain 1/2 in cardiac myocytes contributes to decreased contractility during apoptosis before overt cell death. Reduced myosin abundance has also been observed in the cardiomyocyte cell line H9c2 after hypoxia–reoxygenation. Moreover, overexpression of myosin 1b reversed hypoxia–reoxygenation‐induced apoptosis by increasing antiapoptotic Bcl‐2 and decreasing pro‐apoptotic Bax and cleaved‐caspase 3 protein levels [51]. Additionally, myosin 1b can interact with PTEN (phosphatase and tensin homolog), preventing its localization in the nucleus and thus favoring nuclear AKT activation and cell survival in various cell types [52]. Conversely, myosin light chains themselves can be targets of caspase‐3: myosin light chain 1 is cleaved by caspase‐3, and its localization in sarcomeres is lost in failing cardiomyocytes, representing a potential molecular mechanism contributing to deterioration of cardiac function before myocyte cell death [53].

The observation that *Pcyox1* silencing was associated with increased abundance of myosins in the mitochondrial fraction warrants discussion. Myosin localization to mitochondria has been previously demonstrated. For instance, myosin 19 has been implicated in mitochondrial dynamics and function [54, 55]. These observations support the concept that specific actin–myosin systems can interface with mitochondrial organization and dynamics.

In *Pcyox1*‐deficient cells, we observed a significant reduction in the levels of the protein S100‐A11 (also referred to as S100C and calgizarin), an EF hand‐type Ca^2+^‐binding protein implicated in cell growth, inflammation, and cytoskeleton dynamics [56]. S100‐A11 has been identified as a new regulator of aldosterone‐induced collagen production in human cardiac fibroblasts and a mediator of cardiac fibrosis [57]. Increased expression of S100A11 has also been reported in cardiomyocytes of rat hearts treated with isoproterenol, suggesting a potential involvement in isoproterenol‐induced myocardial damage [58].

## 5 Conclusions

In conclusion, this study identifies PCYOX1 as a contributor to cardiomyocyte redox homeostasis under hypoxia–reoxygenation stress. In HL‐1 cells, *Pcyox1* silencing was associated with reduced ROS accumulation, decreased apoptosis, and proteomic remodeling involving energy metabolism, contractile machinery, and stress‐responsive pathways. These data support a role for PCYOX1 as a modulator—and potentially an amplifier—of oxidative stress during ischaemic‐like insults, whereas acknowledging that additional ROS‐producing systems may contribute depending on context. Although HL‐1 cells provide a robust mechanistic model, future validation in human iPSC‐derived cardiomyocytes and in vivo systems will be necessary to establish physiological relevance. Collectively, PCYOX1 emerges as a candidate target to mitigate oxidative damage in cardiac cells, warranting further mechanistic and translational investigation.

## Author Contributions

C. Banfi and S. Eligini conceived and designed the research. A. Mallia, L. Brocca, A. Bascucci, and G.G. Papaianni performed the research and acquired the data. C. Banfi, A. Mallia, and S. Eligini analyzed and interpreted the data. All authors were involved in drafting and revising the manuscript.

## Funding

This research was funded by the European Union—Next Generation EU—NRRP M6C2—Investment 2.1 Enhancement and strengthening of biomedical research in the NHS—PNRR‐POC‐2023‐12377609—B43C24000320006.

## Consent

The authors have nothing to report.

## Conflicts of Interest

We disclose the following European Patent application, under examination, EP15817414.4 entitled “Prenylcysteine oxidase 1 inhibitors for the prevention and/or treatment of oxidative stress‐related degenerative diseases and prenylcysteine oxidase 1 as diagnostic marker,” applicant Centro Cardiologico Monzino IRCCS, with no financial conflicts of interest. The remaining authors declare no conflicts of interest.

## Supporting information


**Figure S1:** Expression of pro‐inflammatory mediators in HL‐1 *Pcyox1* silenced cells.

**Table S1:** Proteins modulated by *Pcyox1* silencing in ischaemic/hypoxic stress condition.

**Table S2:** Proteins modulated by *Pcyox1* silencing after reoxygenation.

## Data Availability

Data collected in the study will be made available using the data repository Zenodo (https://zenodo.org/records/18984137). Proteomic data are available via ProteomeXchange with identifier PXD040095 and PXD040093. Any remaining information can be obtained from the corresponding author upon reasonable request.
