# Supplementary material for: Prenylcysteine Oxidase 1 Deficiency Protects the Cardiac Muscle Cell Line HL‐1 Against Ischaemic/Hypoxic Stress
Source: FASEB J. 2026 Apr 20;40:e71819. doi: 10.1096/fj.202502993R (PMC13094462; doi:10.1096/fj.202502993R)
Supplement: Supplementary file 1 — Figure S1: Expression of pro‐inflammatory mediators in HL‐1 Pcyox1 silenced cells. [file FSB2-40-e71819-s002.pptx]

## Slide 1
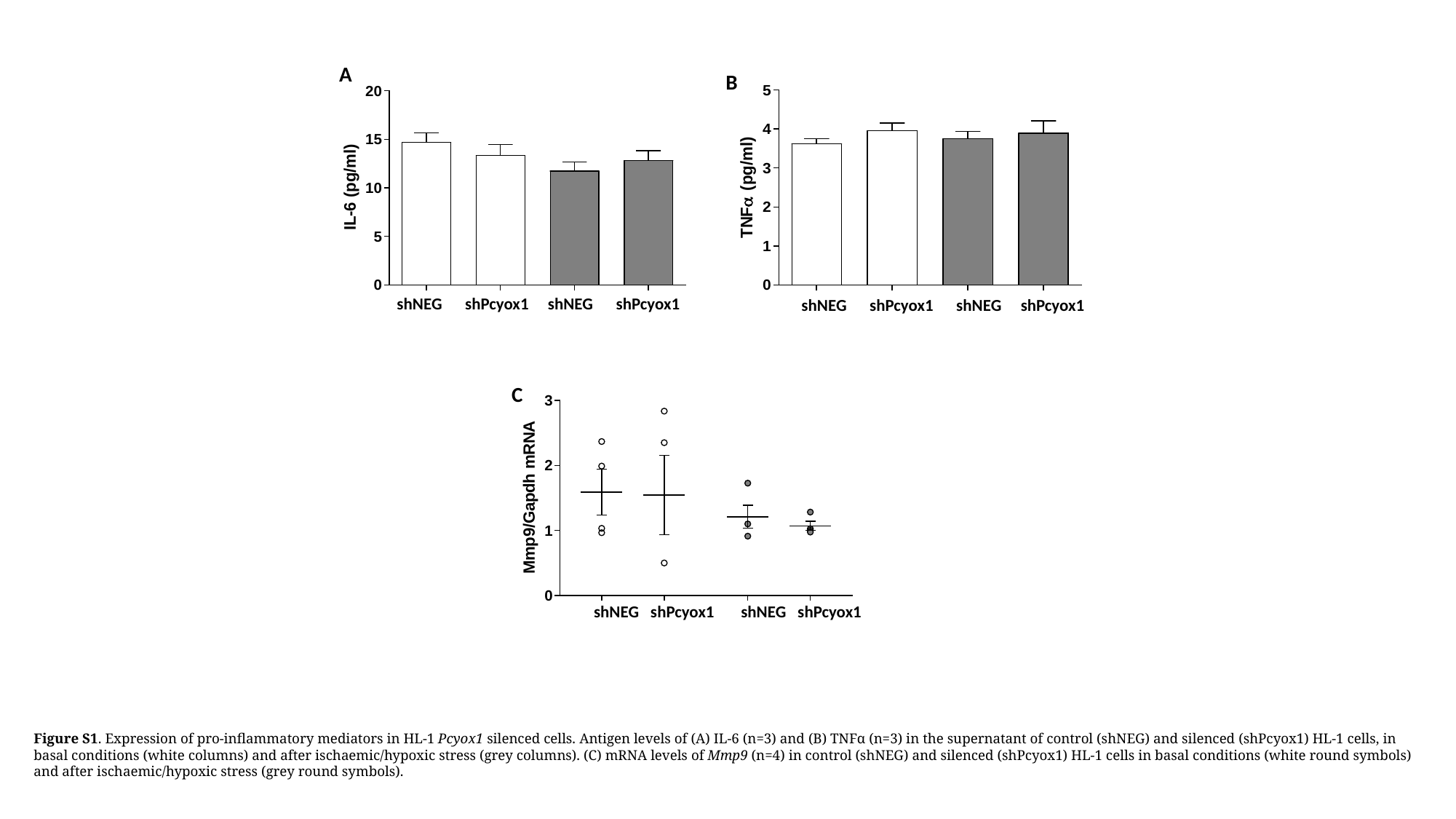

A
shNEG shPcyox1 shNEG shPcyox1
shNEG shPcyox1 shNEG shPcyox1
B
shNEG shPcyox1 shNEG shPcyox1
C
Figure S1. Expression of pro-inflammatory mediators in HL-1 Pcyox1 silenced cells. Antigen levels of (A) IL-6 (n=3) and (B) TNFα (n=3) in the supernatant of control (shNEG) and silenced (shPcyox1) HL-1 cells, in basal conditions (white columns) and after ischaemic/hypoxic stress (grey columns). (C) mRNA levels of Mmp9 (n=4) in control (shNEG) and silenced (shPcyox1) HL-1 cells in basal conditions (white round symbols) and after ischaemic/hypoxic stress (grey round symbols).
