# Supplementary material for: Prenylcysteine Oxidase 1 Deficiency Protects the Cardiac Muscle Cell Line HL‐1 Against Ischaemic/Hypoxic Stress
Source: FASEB J. 2026 Apr 20;40:e71819. doi: 10.1096/fj.202502993R (PMC13094462; doi:10.1096/fj.202502993R)
Supplement: Supplementary file 2 — Table S1: Proteins modulated by Pcyox1 silencing in ischaemic/hypoxic stress condition. [file FSB2-40-e71819-s003.docx]

**Table S1**. Proteins modulated by *Pcyox1* silencing in ischaemic/hypoxic stress condition

| **Accession** | **Unique peptides** | **Score** | **Anova (p)** | **Max fold change** | **Description** |
| --- | --- | --- | --- | --- | --- |
| **Increased with *Pcyox1* silencing under hypoxia** | | | | |  |
| P05132 | 4 | 56.18 | 0.020902 | **1.57** | cAMP-dependent protein kinase catalytic subunit alpha OS=Mus musculus GN=Prkaca PE=1 SV=3 |
| P30275 | 5 | 65.86 | 1.89E-05 | **1.47** | Creatine kinase U-type_ mitochondrial OS=Mus musculus GN=Ckmt1 PE=1 SV=1 |
| P46660 | 2 | 38.85 | 0.009043 | **2.08** | Alpha-internexin OS=Mus musculus GN=Ina PE=1 SV=3 |
| Q6P8J7 | 5 | 58.2 | 5.14E-08 | **2.73** | Creatine kinase S-type_ mitochondrial OS=Mus musculus GN=Ckmt2 PE=1 SV=1 |
| Q9JI91 | 9 | 229.19 | 0.001474 | **1.31** | Alpha-actinin-2 OS=Mus musculus GN=Actn2 PE=1 SV=2 |
| Q9QVP4 | 8 | 135.14 | 0.002966 | **1.50** | Myosin regulatory light chain 2_ atrial isoform OS=Mus musculus GN=Myl7 PE=1 SV=1 |
| Q9WUB3 | 5 | 258.08 | 0.000436 | **1.54** | Glycogen phosphorylase_ muscle form OS=Mus musculus GN=Pygm PE=1 SV=3 |
| Q02566 | 21 | 1040.69 | 2.54E-05 | **1.79** | Myosin-6 OS=Mus musculus GN=Myh6 PE=1 SV=2 |
| **Decreased with *Pcyox1* silencing under hypoxia** | | | | | |
| O88792 | 3 | 60.6 | 0.000475 | **1.85** | Junctional adhesion molecule A OS=Mus musculus GN=F11r PE=1 SV=2 |
| P26350 | 6 | 51.3 | 4.61E-05 | **1.61** | Prothymosin alpha OS=Mus musculus GN=Ptma PE=1 SV=2 |
| P28667 | 3 | 39.24 | 0.000722 | **1.69** | MARCKS-related protein OS=Mus musculus GN=Marcksl1 PE=1 SV=2 |
| P50543 | 2 | 13.58 | 5.28E-05 | **3.03** | Protein S100-A11 OS=Mus musculus GN=S100a11 PE=1 SV=1 |
| P52293 | 7 | 69.02 | 0.000696 | **1.52** | Importin subunit alpha-1 OS=Mus musculus GN=Kpna2 PE=1 SV=2 |
| Q9D662 | 2 | 47.69 | 0.000867 | **2.16** | Protein transport protein Sec23B OS=Mus musculus GN=Sec23b PE=1 SV=1 |
| Q9Z0P4 | 2 | 27.67 | 0.002029 | **3.04** | Paralemmin-1 OS=Mus musculus GN=Palm PE=1 SV=1 |
| Q91VK4 | 2 | 12.65 | 0.005422 | **3.12** | Integral membrane protein 2C OS=Mus musculus GN=Itm2c PE=1 SV=2 |
| Q922Q1 | 3 | 19.52 | 0.000336 | **1.62** | Mitochondrial amidoxime reducing component 2 OS=Mus musculus GN=Marc2 PE=1 SV=1 |
| Q64337 | 9 | 60.38 | 0.000242 | **1.91** | Sequestosome-1 OS=Mus musculus GN=Sqstm1 PE=1 SV=1 |
