# Supplementary material for: Prenylcysteine Oxidase 1 Deficiency Protects the Cardiac Muscle Cell Line HL‐1 Against Ischaemic/Hypoxic Stress
Source: FASEB J. 2026 Apr 20;40:e71819. doi: 10.1096/fj.202502993R (PMC13094462; doi:10.1096/fj.202502993R)
Supplement: Supplementary file 3 — Table S2: Proteins modulated by Pcyox1 silencing after reoxygenation. [file FSB2-40-e71819-s001.docx]

**Table S2.** Proteins modulated by *Pcyox1* silencing after reoxygenation.

| **Accession** | **Unique peptides** | **Score** | **Anova (p)** | **Max fold change** | **Description** |
| --- | --- | --- | --- | --- | --- |
| **Increased with *Pcyox1* silencing after hypoxia and reoxygenation** | | | | | |
| O70468 | 11 | 112.1 | 0.000431 | **1.50** | Myosin-binding protein C_ cardiac-type OS=Mus musculus GN=Mybpc3 PE=1 SV=1 |
| P05132 | 4 | 56.2 | 4.31E-05 | **2.02** | cAMP-dependent protein kinase catalytic subunit alpha OS=Mus musculus GN=Prkaca PE=1 SV=3 |
| P31324 | 5 | 60.7 | 0.002349 | **2.39** | cAMP-dependent protein kinase type II-beta regulatory subunit OS=Mus musculus GN=Prkar2b PE=1 SV=3 |
| Q6P8J7 | 5 | 58.0 | 0.000123 | **2.79** | Creatine kinase S-type_ mitochondrial OS=Mus musculus GN=Ckmt2 PE=1 SV=1 |
| Q9CZS1 | 7 | 122.1 | 0.004311 | **1.58** | Aldehyde dehydrogenase X_ mitochondrial OS=Mus musculus GN=Aldh1b1 PE=1 SV=1 |
| Q9D023 | 5 | 37.7 | 0.000334 | **1.43** | Mitochondrial pyruvate carrier 2 OS=Mus musculus GN=Mpc2 PE=1 SV=1 |
| Q9ES82 | 4 | 30.7 | 0.000268 | **1.72** | Popeye domain-containing protein 2 OS=Mus musculus GN=Popdc2 PE=1 SV=1 |
| Q9QVP4 | 8 | 135.1 | 1.70E-05 | **1.70** | Myosin regulatory light chain 2_ atrial isoform OS=Mus musculus GN=Myl7 PE=1 SV=1 |
| Q02566 | 21 | 1040.7 | 5.92E-06 | **1.96** | Myosin-6 OS=Mus musculus GN=Myh6 PE=1 SV=2 |
| **Decreased with *Pcyox1* silencing after hypoxia and reoxygenation** | | | | | |
| P50543 | 2 | 13.6 | 2.75E-05 | **2.61** | Protein S100-A11 OS=Mus musculus GN=S100a11 PE=1 SV=1 |
| O88792 | 3 | 60.6 | 0.000803 | **1.76** | Junctional adhesion molecule A OS=Mus musculus GN=F11r PE=1 SV=2 |
| P28667 | 3 | 39.2 | 0.002287 | **1.52** | MARCKS-related protein OS=Mus musculus GN=Marcksl1 PE=1 SV=2 |
| P47738 | 8 | 116.1 | 0.000139 | **1.50** | Aldehyde dehydrogenase_ mitochondrial OS=Mus musculus GN=Aldh2 PE=1 SV=1 |
| P63260 | 7 | 326.3 | 0.002486 | **1.35** | Actin_ cytoplasmic 2 OS=Mus musculus GN=Actg1 PE=1 SV=1 |
| Q91VK4 | 2 | 12.6 | 0.002269 | **1.86** | Integral membrane protein 2C OS=Mus musculus GN=Itm2c PE=1 SV=2 |
| Q922Q1 | 3 | 19.5 | 0.001671 | **1.58** | Mitochondrial amidoxime reducing component 2 OS=Mus musculus GN=Marc2 PE=1 SV=1 |
| Q64337 | 9 | 60.4 | 0.000277 | **1.88** | Sequestosome-1 OS=Mus musculus GN=Sqstm1 PE=1 SV=1 |
|  |  |  |  |  |  |
